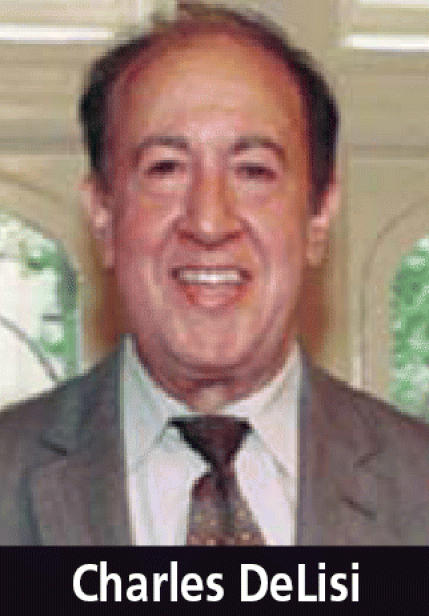# Guest Editorial: Systems Biology, the Second Time Around

**DOI:** 10.1289/ehp.112-1247662

**Published:** 2004-11

**Authors:** Charles DeLisi

**Affiliations:** Bioinformatics Program, Boston University, Boston, MA, E-mail: delisi@bu.edu

When T.S. Eliot (Eliot 1963) wrote “. . . the end of all our exploring Will be to arrive where we started And know the place for the first time,” he was probably not thinking explicitly of science, but as science is deeply imbedded in the human condition, we should not be surprised that these words ring true.

Systems biology as a quantitative science dates at least to Hermann von Helmholtz, the 19th century German physicist whose studies of metabolism led to the first law of thermodynamics. Helmholtz explored human physiology in its entirety, making fundamental contributions to audition, vision, the conduction of the nervous impulse and, perhaps most important in so far as systems biology is concerned, physiologic energy balance. Our understanding of physiologic systems has of course evolved substantially during the past 150 years, and today sophisticated, if domain-specific, mathematical models are used to simulate, plan, and interpret experiments in numerous branches of biomedicine including endocrinology, cardiovascular physiology, immunology, neurophysiology, and the cognitive sciences. Moreover, with the completion of the first phase of the visible human project, which provides high-resolution MR (magnetic resonance) and CT (computed tomography) imaging scans of male and female anatomies, we can seriously contemplate coupling organ-level models that integrate anatomical, biophysical, and physiologic data to produce a computer-based virtual human.

Molecules are not currently the building blocks of useful organ-level models. Instead, the cell is modeled at low resolution, if not as a black box. For example, a model of the humoral immune response might include B-cell trafficking, stimulation by antigen, and regulation by T cells. The dynamics of helper and suppressor T cells and their interaction with antigen-presenting cells could be modeled as a separate subsystem, or module, whose output served as input to the B-cell module. The response of B cells to antigen would then be modeled using experimentally determined rate constants for antigen–receptor interaction to obtain receptor occupancy, and a phenomenologic function determined experimentally would relate occupancy and T-cell state to antibody secretion and B-cell proliferation rate.

The levels of depth that would not be modeled explicitly are apparent. The antigen–receptor rate constants could themselves in principle be calculated in terms of the detailed atomic-level structures of the antigen and immunoglobulin receptors, using long-and short-range force fields determined by quantum chemical calculations and thermodynamic measurements. Such calculations, even if crystal structures were available and the force fields were known precisely, would need to take into account conformational rearrangements in surface side chains, some backbone adaptation, and solvent restructuring. Such calculations are currently too difficult to perform routinely with even moderate precision.

Similarly, one could in principle model by any number of methods—physical chemical, probabilistic, etc.—the signaling pathways leading from receptor occupancy to gene activation, with all the various post-translational modifications and their dependence on the state of the cell, terminating in the modulation of sets of genes combinatorially regulated by sets of transcription factors. But the information required is currently far too sketchy for detailed cell-based models to be useful inputs to organ-and tissue-level models. The advantages of including such deeper-level models explicitly would be *a*) the connection they may provide between the (dynamic) state of the cell’s surface and the gene–protein–metabolite network topology in the interior of the cell, thus providing an entrée to a global-integrated model; *b*) their ability to integrate cell physiology with cell anatomy—just as a virtual human would integrate anatomy and physiology at the organ level; and *c*) the foundation they would provide for deep design; that is, for rational molecular manipulations aimed at production of prespecified phenotypes.

Although historical and global perspectives remind us that we are not in an entirely new place, profound changes have occurred in recent years—changes that are driving a fundamental shift in the culture and content of the life sciences. One such change is, of course, genomic decoding—work that has only just begun. The next 5–10 years will see the production of complete lists of parts of eukaryotic cells, and the next 15–20 years will see reasonably complete wiring diagrams. But—a worn analogy not withstanding—understanding a cell from its list of parts is far more complex than understanding a Boeing 747 airplane or many other complex systems. The cell is not hard wired, therefore a “wiring diagram” only provides, after much analysis, a combinatorially rich repertoire of circuit modules, particular subsets of which are selected by particular environments. And because a cell’s environment is in fugue, the problem of systems biology is understanding the rules of subset selection, and connecting recurrent functional modules to phenotype.

There are many ways to carry out such a program at various levels of spatial and temporal resolution. The level selected depends on experimental or clinical goals. But regardless of the approach used, connecting the genomic revolution and a biology that would understand the cell as a hierarchical system of environmentally selected functional modules is a long-term program. Along the way, as our understanding deepens and as our models attain broader phenomenologic coverage, we can expect to attain a greatly accelerated understanding of evolutionary and developmental biology and greater precision in identifying drug targets and individualizing therapies.

While genomics—and I use the word canonically—does not in itself enable a cell systems biology, it is providing the tools and data that embolden us to begin thinking and working seriously toward that goal. But it is doing much more. It has married the two most powerful technologies of the 20th century—computer science and molecular biology. Computer science is the *sine qua non* for postgenomic biology, and the dexterity with which its leaders have responded to the biological challenge is one of the great stories in the sociology of science. Nevertheless, the fundamental cultural challenge remains with the biology community itself . The pace of progress will continue to be rate limited by the ability of our universities to educate a new generation of biologists. Not an easy task for organizations that—for some good and some not so good reasons—remain instinctively conservative, even as they sow the seeds of revolution.

## Figures and Tables

**Figure:**